# Mid-term subclinical myocardial injury detection in patients who recovered from COVID-19 according to pulmonary lesion severity

**DOI:** 10.3389/fcvm.2022.950334

**Published:** 2022-10-18

**Authors:** Ikram Chamtouri, Rania Kaddoussi, Hela Abroug, Mabrouk Abdelaaly, Taha Lassoued, Nesrine Fahem, Saoussen Cheikh'Hmad, Asma Ben Abdallah, Walid Jomaa, Khaldoun Ben Hamda, Faouzi Maatouk

**Affiliations:** ^1^Cardiology B Department, Hôpital Universitaire Fattouma Bourguiba, Monastir, Tunisia; ^2^Pneumology Department, University of Monastir, Monastir, Tunisia; ^3^Preventive Medicine Department, Hospital Fatuma Bourguiba, Monastir, Tunisia; ^4^Radiology Department, Fattouma Bourguiba University Hospital, Monastir, Tunisia

**Keywords:** coronavirus, strain, cardiac, injury, recovery

## Abstract

**Background:**

Severe acute respiratory syndrome coronavirus 2 (SARS-CoV 2) may cause damage to the cardiovascular system during the acute phase of the infection. However, recent studies reported mid- to long-term subtle cardiac injuries after recovering from acute coronavirus disease 2019 (COVID-19). This study aimed to determine the relationship between the severity of chest computed tomography (CT) lesions and the persistence of subtle myocardial injuries at mid-term follow-up of patients who recovered from COVID-19 infection.

**Methods:**

All patients with COVID-19 were enrolled prospectively in this study. Sensitive troponin T (hsTnT) and chest CT scans were performed on all patients during the acute phase of COVID-19 infection. At the mid-term follow-up, conventional transthoracic echocardiograph and global longitudinal strain (GLS) of the left and right ventricles (LV and RV) were determined and compared between patients with chest CT scan lesions of < 50% (Group 1) and those with severe chest CT scan lesions of greater or equal to 50% (Group 2).

**Results:**

The mean age was 55 ± 14 years. Both LV GLS and RV GLS values were significantly decreased in group 2 (*p* = 0.013 and *p* = 0.011, respectively). LV GLS value of more than −18 was noted in 43% of all the patients, and an RV GLS value of more than −20 was observed in 48% of them. The group with severe chest CT scan lesions included more patients with reduced LV GLS and reduced RV GLS than the group with mild chest CT scan lesions [(G1:29 vs. G2:57%, *p* = 0.002) and (G1:36 vs. G2:60 %, *p* = 0.009), respectively].

**Conclusion:**

Patients with severe chest CT scan lesions are more likely to develop subclinical myocardial damage. Transthoracic echocardiography (TTE) could be recommended in patients recovering from COVID-19 to detect subtle LV and RV lesions.

## Introduction

Coronavirus disease 2019 (COVID-19) is an emergent infection caused by the new coronavirus severe acute respiratory syndrome coronavirus 2 (SARS-COV 2) (1). Since its appearance, it has caused millions of deaths by affecting several organs.

Although the respiratory system is the most affected, cardiac involvement seems to have a big role in prognosis (2). So, performing transthoracic echocardiography (TTE), especially in critical cases, may help to evaluate prognosis and guide therapeutic management.

Since cardiac injury is frequent in patients with COVID-19, realizing routine standard echocardiography and, especially, obtaining a global longitudinal strain (GLS) measurement is recommended.

Many studies (3, 4) showed the importance of longitudinal strain study in hospitalized patients with confirmed COVID-19 infection. However, only a few researchers have studied its contribution to the detection of sub-clinical myocardial injuries after recovery from acute infection. The subclinical myocardial damage is determined using a myocardial speckle tracking study by measuring the left ventricular (LV) GLS and the right ventricular (RV) GLS (5).

This study aimed to reveal cardiac abnormalities at mid-term follow-up in patients who recovered from COVID-19 by using LV GLS and RV GLS values. It also compared the degree of LV GLS and RV GLS alteration between patients already having severe chest computed tomography (CT) scan lesions ≥ 50% and those with chest CT scan lesions < 50% at the acute phase of infection.

## Methods

### Study design and population

This is a cohort study conducted in the three COVID centers from January 2021 to March 2021.

### Inclusion criteria

We enrolled a cohort of recovered patients from COVID-19 infection from three COVID-19 centers.

Diagnosis of COVID-19 was confirmed by the presence of a real time reverse –transcription polymerase chain reaction (RT-PCR).

### Non-inclusion criteria

Patients who did not agree to participate were not included.

### Exclusion criteria

Patients with pulmonary embolism, a high-sensitivity cardiac troponin (hs-cTn) serum level> the 99th percentile (0.014 ng/mL) (3) during the acute phase of the -COVID-19 infection, a level of hs-cTn level more than the 99th percentile (0.014 ng/mL) at 3 months, poor acoustic windows, severe valvular heart disease, LVEF < 50%, arrhythmia, or coronary artery disease were excluded.

### Variables

All the patients underwent a clinical examination, that is, blood tests including hs-cTn, C-reactive protein, D-dimers, hematocrit, serum creatinine, and N-terminal prohormone of the brain natriuretic peptide (NT-proBNP).

**Figure 1:**
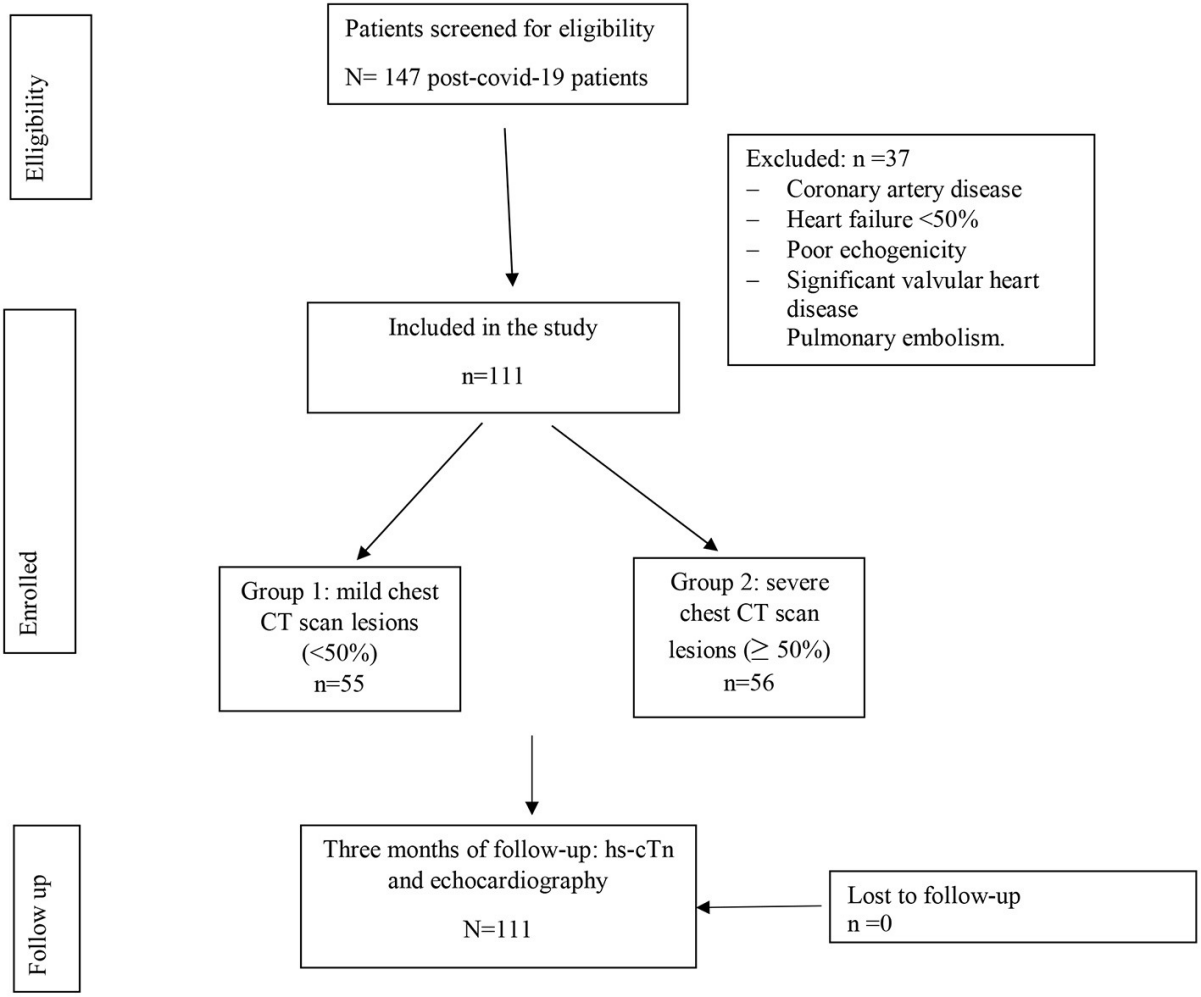
The flow chart of the study population.

A chest CT scan was performed within the first 14 days from the onset of the symptoms. TTE with longitudinal strain study was performed 3 months after recovery (Figure 1).

Patients were divided into two groups. Group 1: those with Chest CT scan lesions < 50% and Group 2: those with chest CT scan lesions equal to or >50%.

### Data sources and measurement

Demographic, biological, and scanner data were collected from patients during hospitalization in the COVID-19 units.

**Figure 2:**
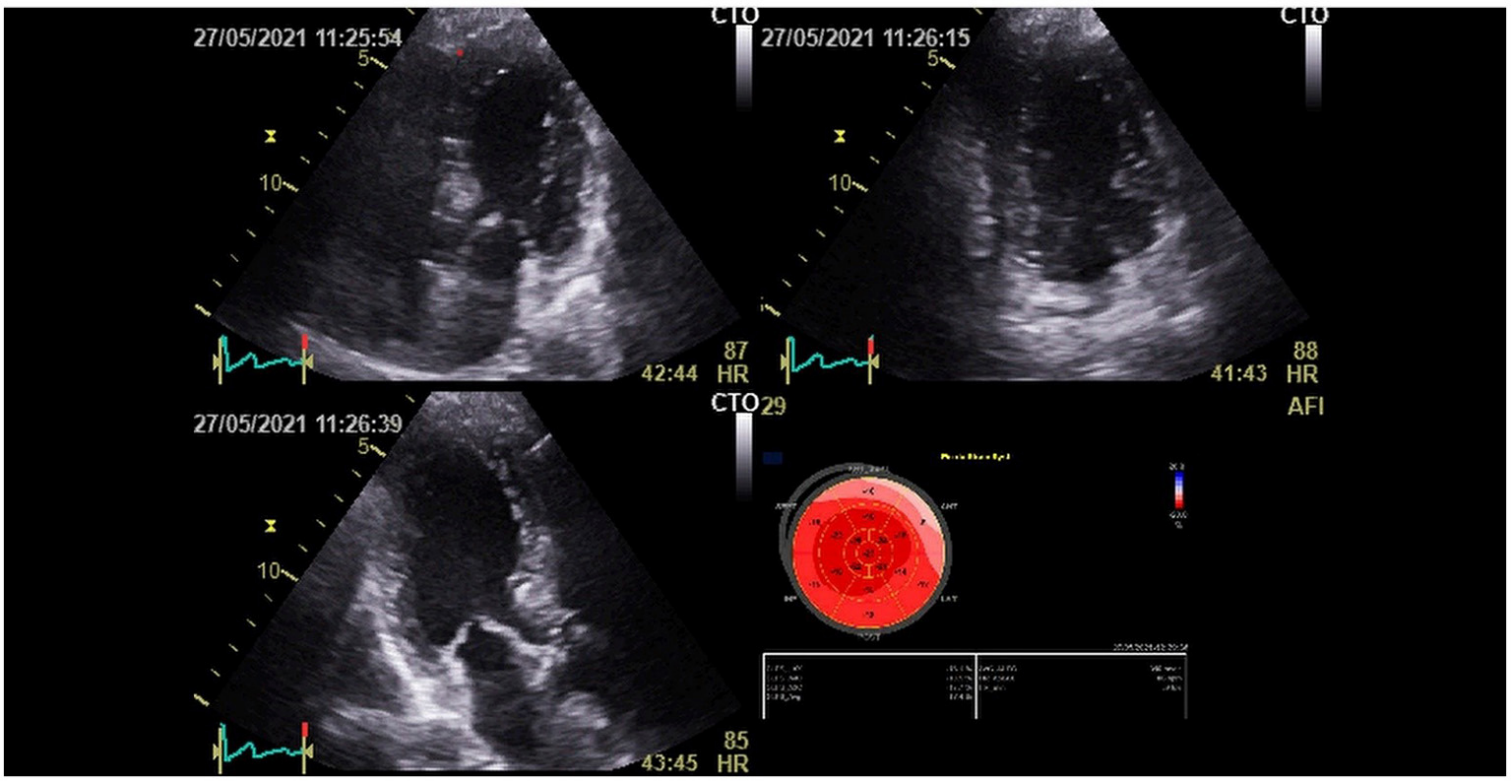
Left ventricular global longitudinal strain (LV GLS) and bull's eye plot recovered patient from the COVID-19 infection.

**Figure 3:**
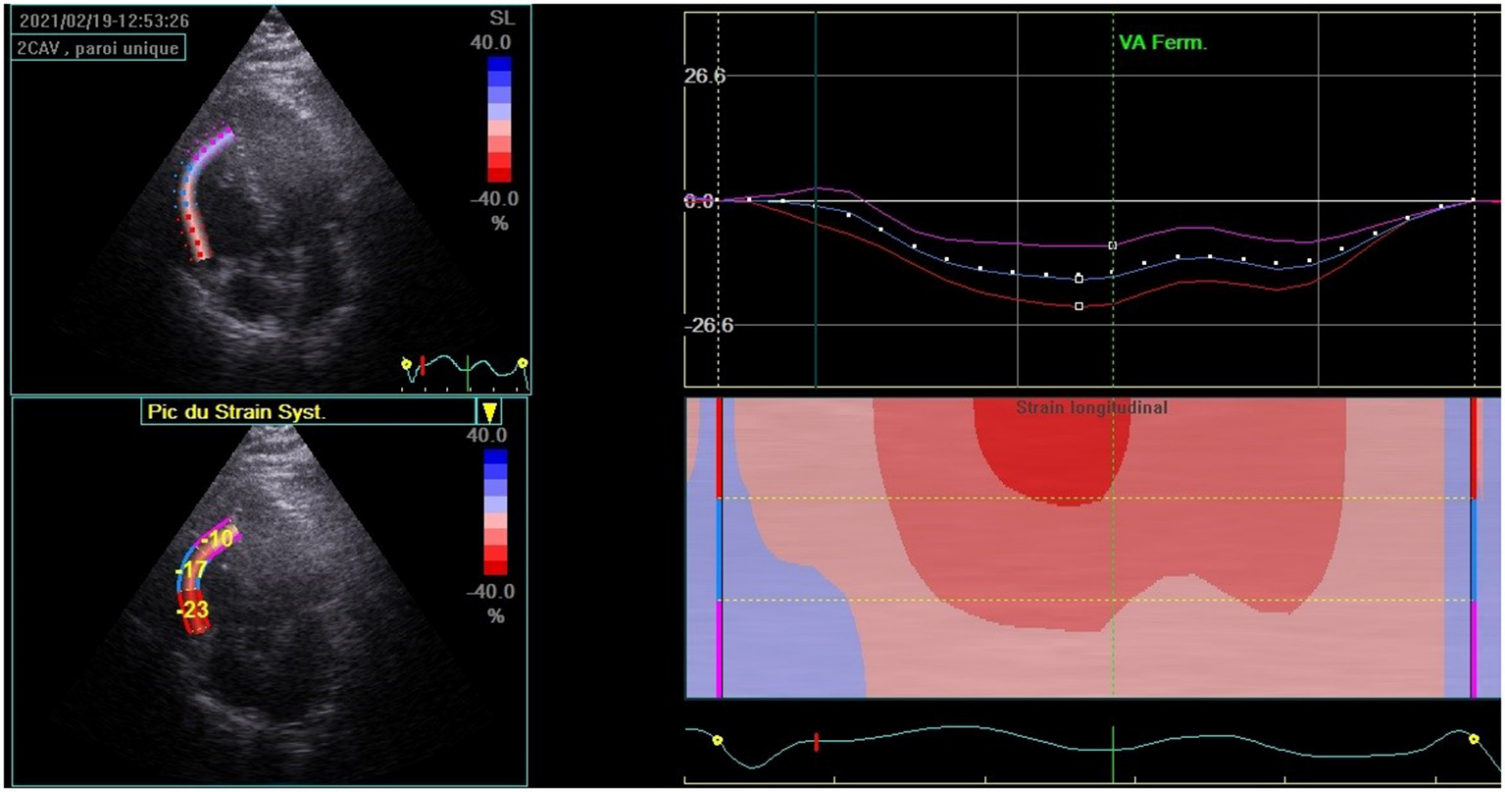
Right ventricular global longitudinal strain (RV GLS) calculated from the mean strain values of the RV three segments (basal, mid, and apical) in patients recovered from the COVID-19 infection.

At 3 months of follow-up, TTE (Vivid E9 General Electric Medical System) was performed on all participants by the same operator. Conventional TTE parameters, including LV end-systolic and end-diastolic diameters (LVESD and LVEDD), interventricular septum (IVS), and LV ejection fraction (LVEF), were measured. Diastolic LV function was evaluated. RV systolic function was measured by using peak right ventricular systolic myocardial velocity (S wave) and tricuspid annular plane systolic excursion (TAPSE). The atrial areas and systolic pulmonary blood pressure (SPBP) were measured. Offline speckle tracking analyses were performed in all participants using Echopac software version 112 and based on apical views of two, three, and four chambers for the LV study. GLS was obtained after tracking the endocardial borders and by calculating the mean peak systolic strain values of the 17 segments. LV GLS and bull's eye plot were obtained (Figure 2). A value of LV GLS of more than −18% was regarded as pathological (4). RV GLS was obtained from the apical four-chambered view by tracking the RV free wall endocardial borders. RV GLS was calculated from the mean strain values of the RV three segments (basal, mid, and apical) (Figure 3). A value of RV GLS of more than −20% was considered pathological (6).

### Statistical analysis

Statistical analysis was performed using Statistical Program for Social Sciences (SPSS) version 20.0. Categorical variables were presented as frequency rates and percentages. The Chi-Square test (χ2 test) or the Fisher exact test were used to compare frequencies. The Kolmogorov–Smirnov test was used to confirm the normal distribution of continuous variables. The normally distributed data were expressed as mean ± standard deviation (SD), and Student's *t*-test was performed to compare means in two independent samples. The non-normally distributed data were presented as median with interquartile range (IQR) and compared by the Mann-Whitney *U*-test. A *p*-value of < 0.05 was considered statistically significant.

### Ethical considerations

The cohort of patients is a part of the research protocol (IORG 00093738 N°102/OMB 0990-0279) approved by the Hospital Ethics Committee of the Faculty of Medicine of Monastir. The study was conducted in accordance with Good Clinical Practice conditions and standard ethical considerations.

All participants had signed informed consent.

## Results

### I-Population's general characteristics

A total of 147 patients were initially enrolled. Of which, 37 were excluded due to the exclusion criteria. Thus, a total of 111 patients were included in the final analysis. A total of 55 patients (49.55%) had mild chest CT scan lesions (group 1) and 56 patients (50.45%) had severe chest CT scan lesions (group 2) as shown in Figure 1.

No significant differences were noted between the two groups regarding all epidemiological characteristics except for smoking status and diabetes mellitus. Compared to group 1, a higher prevalence of ground glass opacity was noted in group 2 (86.2 vs. 94.4%; *p* = 0.05). Baseline data of the overall population and the two groups are presented in Table 1.

**Table 1:** The characteristics, symptoms, comorbidities, and laboratory findings of patients.

**Clinical characteristics**	**COVID-19 (*N =* 111)**	**G1: Mild COVID-19** **(*N =* 55)**	**G2: Severe COVID-19 (*N =* 56)**	***P*-value**
Male (*n***;** %)	64 (57.1%)	28 (51.6%)	38 (66.7%)	0.30
Age (years) (mean ± SD)	62.5 ± 13.9	62 ± 9	61 ± 14	0.70
BMI (Kg/m^2^) (mean ± SD)	29.5 ± 6.3	28.9 ± 5.6	30.8 ± 7.5	0.34
Smoking (*n***;** %)	10 (8.7%)	4 (6.9%)	6 (11.8%)	**0.04**
**Co morbidities**				
Diabetes mellitus (*n***;** %)	35 (31.8%)	14 (26.7%)	21 (37.5%)	**0.031**
Hyperlipidemia (*n*; %)	16 (14.6%)	9 (16.7%)	7 (11.1%)	0.69
Hypertension (*n*; %)	51 (45.8%)	20 (40%)	31 (55.6%)	0.29
Chronic obstructive pulmonary disease (*n*; %)	4 (3.6%)	1 (3.3%)	3 (5.6%)	0.53
**Clinical parameters**				
Oxygen saturation (mean ± SD)	86.3 ± 6.7	88 ± 6.7	84 ± 6.1	0.13
Respiratory rate (min^−1^) (mean ± SD)	23.8 ± 5	23.2 ± 6.7	24.6 ± 4.9	0.33
Heart rate (bpm)	80 [74–90]	80 [72,7–96,25]	80 [72–90]	0.36
Systolic blood pressure (mmHg) (mean ± SD)	122 ± 12	125 ± 14	123 ± 14	0.22
Diastolic blood pressure (mmHg) (mean ± SD)	71 ± 12	74 ± 12	69 ± 11	0.19
**Signs**				
Nausea/vomiting (*n***;** %)	21 (19.1%)	13 (23.3%)	8 (11.8%)	0.45
Fever (*n***;** %)	80 (72.3%)	39 (70%)	41 (76.5%)	0.64
Fatigue (*n***;** %)	73 (66%)	32 (60%)	41 (76.5%)	0.25
Headache (*n***;** %)	33 (29.8%)	14 (26.7%)	19 (35.3%)	0.74
Chest pain (*n***;** %)	21 (19.1%)	9 (16.7%)	12 (23.5%)	0.70
Cough (*n***;**%)	73 (66%)	31 (60%)	42 (76.5%)	0.25
Dyspnea (*n***;**%)	94 (87.2%)	48 (86.7%)	46 (88.2%)	1
**Laboratory data**				
WBC counts (mean ± SD)	8,869 ± 3,755	9,798 ± 3,914	8,491 ± 3,498	0.23
Neutrophil (mean ± SD)	6,997 ± 3,438	6,636 ± 3,194	7,941 ± 3,775	0.21
Lymphocyte (mean ± SD)	1309 ± 632	1,453 ± 6,58	1,256 ± 607	0.30
Albumin(g/L) (mean ± SD)	33 ± 4.7	34 ± 4.5	29 ± 1.4	**0.02**
Creatinine(mmol/L) (mean ± SD)	73 ± 30	69,5 ± 23,4	80 ± 38,6	0.25
Platelet count (x10^3^/μL) (mean ± SD)	264 ± 112	250 ± 111	287 ± 117	0.30
CRP (mg/L) (median [IQR])	54 [17–122]	54 [16–130]	67 [17–118]	0.14
D-dimer (ng/mL) (median [IQR])	621 [358–1,061]	608 [342–989]	535 [352–1,092]	0.81
Aspartate transaminase (U/L) (mean ± SD)	35 ± 18	34 ± 16	37 ± 22	0.58
Alanine transaminase (U/L) (median [IQR])	32 [16–52]	29.5 [17.25–43.5]	29 [15–48]	0.30
**Biomarkers for myocardial injury**				
hs-cTn (ng/mL) (mean ± SD)	0.01 [0.01–0.02]	0.01 [0.01–0.02]	0.02 [0.01–0.04]	0.23
Pro–BNP (pg/mL) (median [IQR])	21 (10–32)	15 [8.7–30.2]	28.5 [12.2–48.2]	0.22
Lactate dehydrogenase (U/L) (mean ± SD)	306 ± 144	294 ± 112	327 ± 195	0.58
Radiological findings (*n =* 56)				
Crazy paving pattern (*n***;** %)	33 (29.8%)	17 (31%)	16 (27.8%)	0.81
Pulmonary consolidation (*n***;** %)	73 (66%)	33 (62.1%)	40 (72.2%)	0.47
Ground glass opacity (*n***;**%)	99 (89.4%)	47 (86.2%)	52 (94.4%)	**0.05**
**Outcomes**				
Hospital stay (days) (mean ± SD)	15 ± 8	11.1 ± 4.3	13.9 ± 7.3	0.19

BMI, body mass index; hs-cTn, high-sensitivity cardiac troponin; NT-proBNP, N-Terminal prohormone of Brain Natriuretic Peptide; SD, standard deviation; WBC, white blood cells.

### II- Echocardiographic characteristics

At 3 months of follow-up, TTE was performed on all participants. All echocardiographic measurements are reported in Table 2. LVEF was normal in the two groups. Compared to group 1, both mean LV GLS and RV GLS were significantly decreased in group 2 (*p* = 0.013 and *p* = 0.011, respectively). Group 2 included more patients with reduced LV GLS (65.2 vs. 42.4%; *p* = 0.07) and RV GLS (82.6 vs. 42%, *p* = 0.003) compared to group 1.

**Table 2:** Echocardiography and myocardial strain measurement in patients after COVID-19.

	**General population (*N =* 111)**	**Group 1** **(*N =* 55)**	**Group 2 (*N =* 55)**	***P*-value**
LVEF (%) (mean ± SD)	64.2 ± 6.1	64.4 ± 6.4	63.7 ± 5.8	0.7
LVEDD (mm) (mean ± SD)	44.8 ± 5.3	44.5 ± 5.8	45.5 ± 4.3	0.5
LVESD (mm) (mean ± SD)	29.08 ± 4.9	28.4 ± 5	30.4 ± 4.8	0.2
IVS (mm) (mean ± SD)	10.3 ± 1.7	10.2 ± 1.8	10.6 ± 1.3	0.5
TAPSE (mean ± SD)	23.5 ± 4.1	22.6 ± 3.8	24.6 ± 4.5	0.14
LV GLS (mean ± SD)	−17.6 ± 3.4	−17.9 ± 2.9	−16 ± 3.7	**0.013**
RV GLS (mean ± SD)	−20.1 ± 4.6	−20.6 ± 4.5	−18.2 ± 3.4	**0.011**
LVGLS ≤ -18 (*n*; %)	59(51.8%)	23 (42.4%)	36 (65.2%)	0.07
RV GLS ≤ -20 (*n*; %)	67(58.9%)	22 (42%)	45 (82.6%)	**0.003**

IVS, inter-ventricular septum thickness; LV, Left ventricle; LVEDD, Left ventricle end-diastolic diameter; LVEF, left ventricle ejection fraction; LVESD, Left ventricle end-systolic diameter; GLS, global longitudinal strain; RV, right ventricular; TAPSE, tricuspid annular.

## Discussion

Patients with COVID-19 showed myocardial involvement that could be due to SARS-CoV-2 virus lesions or could be a sequela of myocardial inflammation. Myocardial involvement induced by SARS-CoV-2 infection is an important indicator for long-term prognosis.

The present study aimed to determine residual cardiac injury at mid-term follow-up among hospitalized patients who recovered from COVID-19 and had no biological stigmata of myocardial injury, according to the severity of the previous pulmonary lesion. The results showed that, compared to group 1(mild chest CT scan lesions), both mean LV GLS and RV GLS were significantly decreased in the patients with severe chest CT scan lesions compared to those with no severe pulmonary lesions (*p* = 0.013 and *p* = 0.011, respectively).

To the best of our knowledge, this is the first study evaluating the relationship between biventricular strain in patients who recovered from COVID-19 and lung injury severity. According to a previous study, cardiovascular injuries due to COVID-19 infection could be manifested by acute coronary syndrome, myocarditis, arrhythmia, hypertension, and pericarditis (2, 7–9). Increased troponin levels during the acute phase of COVID-19 infection could define myocardial injury or other extra cardiac anomalies such as renal failure (10, 11). A cardiac injury may be explained by the direct impact of the virus on myocardial tissue, hypercoagulation and thrombosis, endothelial dysfunction, and high cardiac stress due to hypoxemia and myocardial inflammation (5, 12). In this study, the elevated troponin level at the acute phase and mid-term follow-up was an exclusion criterion.

Previous studies showed that preserved LVEF in patients with COVID-19 is associated with a decreased LV GLS, suggestive of occult myocardial injury, which is a marker of poor prognoses such as heart failure and mortality (13). Despite eventually associated comorbidities like hypertension or diabetes, which may disrupt LV GLS results, LV GLS has been demonstrated to be an independent predictor factor of bad outcomes in patients with COVID-19 (14). It could be reduced even in normotensive and non-diabetic patients (15, 16). Previous studies evaluated the correlation between LV GLS, COVID-19 symptoms, BNP, and troponin levels (17). However, the present study showed the correlation between the impact of lung injury and the severity of LV GLS alteration 3 months after recovering from the COVID-19 infection. Reduced RV GLS has been demonstrated as a bad prognostic factor in patients with pulmonary embolism, pulmonary hypertension, and acute respiratory distress syndrome (18–20). In patients with COVID-19, RV GLS alteration could be explained by RV inflammation and overload due to pulmonary embolism (21). Several studies reported that reduced RV GLS is associated with poor outcomes (22, 23). In patients with COVID-19, RV free wall speckle tracking has a better prognostic value than the total RV strain (involving inter-ventricular septum), which depends on LV systolic function and motion (14, 24, 25). The current study showed a decrease in RV GLS compared to the reference value, with a significant reduction in the alteration of RV GLS according to the severity of the lung lesions. The potential hypothesis generated from RVGLS alteration may be the concomitant factors inducing injury in both RV and lungs, including the higher inflammatory burden, hypoxemia, and ventilation-induced injury, leading to subclinical RV injury out of the pulmonary embolism context (5, 26).

### Study limitations

There are some limitations to our study. The first limitation is that the three-dimension echocardiography and circumferential and radial strain were not performed. The second limitation is that the echocardiography was not performed during the acute phase of COVID-19 infection. Moreover, cardiac magnetic resonance imaging and cardiac CT imaging were not performed.

## Conclusion

This study showed that patients with severe CT scan pulmonary lesions were more likely to develop sub-clinical myocardial damage at mid-term follow-up, despite the absence of biological stigmata of myocardial injury. Early detection of sub-clinical myocardial dysfunction with speckle tracking echocardiography could be beneficial to ensure early management in patients recovered from COVID-19 infection. Further studies are required to determine the prognostic value of myocardial strain and the long-term evolution of this subclinical myocardial dysfunction in patients after COVID-19.

## Data availability statement

The original contributions presented in the study are included in the article/supplementary material, further inquiries can be directed to the corresponding author/s.

## Ethics statement

The studies involving human participants were reviewed and approved by khochtali Ines. The Ethics Committee waived the requirement of written informed consent for participation.

## Author contributions

IC, RK, HA, and TL were actively involved in data collection and processing. IC, NF, and MA were involved in manuscript preparation. AB, SC, KB, and FM were involved in manuscript reviewing. All authors contributed to the article and approved the submitted version.

## Conflict of interest

The authors declare that the research was conducted in the absence of any commercial or financial relationships that could be construed as a potential conflict of interest.

## Publisher's note

All claims expressed in this article are solely those of the authors and do not necessarily represent those of their affiliated organizations, or those of the publisher, the editors and the reviewers. Any product that may be evaluated in this article, or claim that may be made by its manufacturer, is not guaranteed or endorsed by the publisher.

## Abbreviations

BMI: body mass index
CT: computed tomography
hs-cTn: high-sensitivity cardiac troponin
IVS: interventricular septum
LV: left ventricle
LVEF: LV ejection fraction
LVEDD: LV end-diastolic diameter
LVEDV: LV end-diastolic volume
LV GLS: left ventricle global longitudinal strain
LVESD: LV end-systolic diameter
LVESV: LV end-systolic volume
NT-proBNP: N-Terminal prohormone of Brain Natriuretic Peptide
RV: right ventricle
RV GLS: right ventricle global longitudinal strain
RT-PCR: real time reverse –transcription polymerase chain reaction
TAPSE: tricuspid annular plane systolic excursion
TTE: transthoracic echocardiography.
